# NAD^+^ Enhancer Nicotinamide Riboside Alters Extracellular Purine Metabolism in Human Endothelial Cells

**DOI:** 10.3390/ijms27073267

**Published:** 2026-04-03

**Authors:** Gabriela Harasim-Krawcewicz, Paulina Mierzejewska, Ada Kawecka, Marika A. Frańczak, Oliwia Król, Małgorzata Presler, Agata Jędrzejewska, Barbara Kutryb-Zając, Ryszard T. Smoleński, Ewa Słomińska

**Affiliations:** 1 Department of Biochemistry, Medical University of Gdansk, Debinki 1 Street, 80-211 Gdansk, Poland; gabriela.harasim@gumed.edu.pl (G.H.-K.); paulina.mierzejewska@gumed.edu.pl (P.M.); ada.kawecka@gumed.edu.pl (A.K.); marika.franczak@gumed.edu.pl (M.A.F.); oliwia.krol@gumed.edu.pl (O.K.); malgorzata.presler@gumed.edu.pl (M.P.); agata.jedrzejewska@gumed.edu.pl (A.J.); barbara.kutryb-zajac@gumed.edu.pl (B.K.-Z.); 2 Centre of Experimental Cardiooncology, Medical University of Gdansk, Smoluchowskiego 17 Street, 80-211 Gdansk, Poland

**Keywords:** nicotinamide riboside, NAD metabolism, endothelial cells, purinergic signaling, CD39/CD73 ectonucleotidases, adenosine deaminase, leukocyte adhesion, vascular homeostasis

## Abstract

Nicotinamide adenine dinucleotide (NAD^+^) is essential for maintaining homeostasis in all types of cells, including endothelium, and depletion of its pool can impair bioenergetics and stress response, contributing to cardiovascular disorders. Nicotinamide riboside (NR) effectively restores the intracellular NAD^+^ pool, supporting endothelial integrity, but the molecular mechanisms remain incompletely elucidated, particularly regarding extracellular adenine nucleotide catabolism, purinergic signaling, and their effects on immune cell adhesion. In this study, we aimed to investigate the effects of NR on intracellular nucleotides, extracellular adenine nucleotide catabolism, and adhesive properties in the cultured murine (H5V) and human (HMEC-1) microvascular endothelial cell line. We demonstrated that NR treatment significantly increased intracellular NAD^+^ concentrations without changes in the energy status of endothelial cells. We also showed that NR treatment accelerated extracellular hydrolysis of ATP and AMP and decreased the rate of adenosine deamination in endothelial cells. Moreover, we observed CD73 activity and adenosine-related reduced adhesion of T-lymphocytes, monocytes and platelets to the NR-treated endothelial monolayer. Our findings highlight a previously unrecognized role of NR in maintaining endothelial homeostasis, showing that NR is not only a potent intracellular NAD^+^ booster in endothelial cells but also affects extracellular nucleotide metabolism in a way that promotes cytoprotective adenosine formation.

## 1. Introduction

Nicotinamide adenine dinucleotide (NAD^+^) is a pivotal nucleotide that contributes to the maintenance of cellular energetic homeostasis, exerting regulatory and signaling functions and serving as a coenzyme in redox reactions [1,2]. Depletion of intracellular NAD^+^ is observed in numerous pathologies, including neurodegenerative and cardiovascular disorders, as well as in age-related functional decline, and is often accompanied by dysregulation of NAD^+^ metabolic pathways [3,4,5]. For these reasons, particularly the link between alternations of NAD^+^ metabolism and aging, NAD^+^ is often referred to as the “molecule of youth”, and its precursors have gained popularity as potential therapeutic strategies [6,7]. Nicotinamide riboside (NR) has been identified as one of the most effective precursors of NAD^+^, as it safely and effectively boosts intracellular NAD^+^ levels [8]. Studies in both animal models and humans have demonstrated that NR supplementation significantly elevates NAD^+^ concentrations in multiple tissues, including skeletal muscle, liver, and brain [8,9]. In preclinical studies, NR improved mitochondrial biogenesis and function [10]. NR supplementation also reduced circulating inflammatory cytokines, indicating its potential anti-inflammatory effects [11].

The endothelium, a dynamic cellular layer that lines blood vessels, plays a crucial role in regulating vascular permeability, leukocyte adhesion, and the balance between pro-inflammatory and anti-inflammatory responses [12,13]. Given that NR has been reported to exert vasoprotective and anti-inflammatory effects, it is plausible that NAD^+^ repletion may affect the pathways critical for endothelial homeostasis [14,15].

In the endothelium, extracellular adenine nucleotide catabolism, particularly the ecto-nucleoside triphosphate diphosphohydrolase-1 (CD39),ecto-5′-nucleotidase (CD73), and ecto-adenosine deaminase (eADA) axis, constitutes a major regulatory mechanism controlling leukocyte adhesion, vascular tone, and inflammatory balance [16,17,18,19,20]. Therefore, modulation of intracellular NAD^+^ metabolism may indirectly affect extracellular purinergic signaling. Notably, CD73 occupies a unique position at the interface of NAD^+^ precursor metabolism and purinergic signaling [21]. Beyond its canonical role in AMP-to-adenosine conversion, CD73 participates in extracellular NAD^+^ precursor processing by converting NMN to NR, supporting intracellular NAD^+^ biosynthesis. This dual functionality raises the question of whether exogenous NR supplementation may alter CD73 activity or expression, potentially reshaping both NAD^+^ salvage and extracellular adenosine generation [14,22].

Because extracellular adenosine production is a key determinant of anti-inflammatory and anti-thrombotic endothelial function [23], understanding whether NAD^+^-boosting strategies modulate this pathway is essential for evaluating the full vascular impact of NR supplementation. Our study aims to investigate the effects of NR on intracellular nucleotide levels, extracellular nucleotide metabolism, and adhesive properties of endothelial cells. Addressing this may provide novel insights into the role of NR in endothelial homeostasis and vascular protection, while also emphasizing the ongoing need for research on the exact mechanisms by which NR supplementation affects cellular metabolism.

## 2. Results

### 2.1. Nicotinamide Riboside Effectively Boosts Intracellular NAD^+^ In Vitro Without Changing the Intracellular Adenine Nucleotide Pool

**Figure 1:**
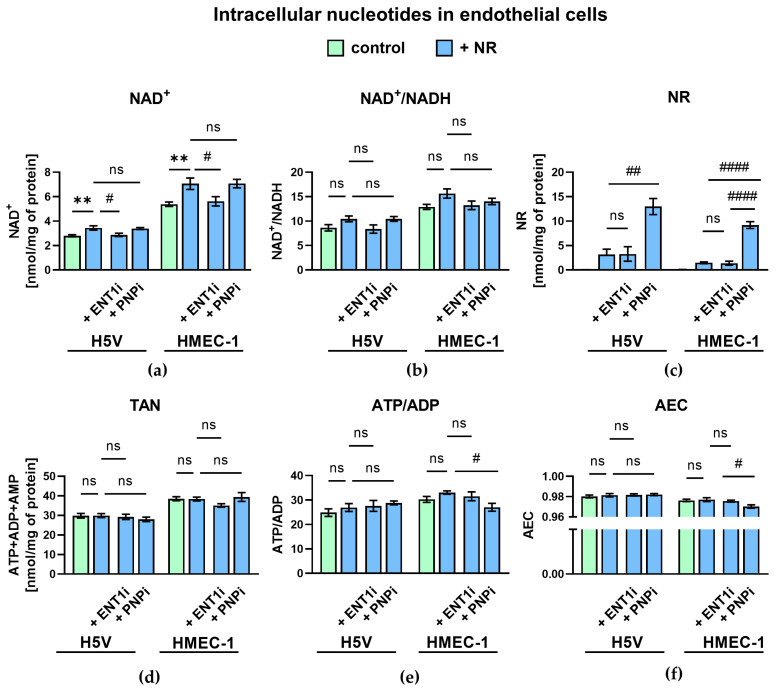
Intracellular nucleotides in NR-treated murine and human endothelial cells. Concentrations of (**a**) NAD^+^, (**b**) NAD^+^/NADH ratio, (**c**) nicotinamide riboside (NR), (**d**) total adenine nucleotides pool (TAN), (**e**) ATP/ADP ratio, and (**f**) adenylate energy charge after 24 h incubation with NR. ENT1i—ENT1 inhibitor (NBTI), PNPi—purine nucleoside phosphorylase inhibitor (forodesine). Results are presented as mean ± SEM, *n* = 3–6; * control vs NR, # NR vs NR + ENT1i, NR vs NR + PNPi, ns—*p* ≥ 0.05, # *p* < 0.05, ** or ## *p* < 0.01, #### *p* < 0.0001; one-way ANOVA and post hoc Holm–Sidak test. Exact *p* values are provided in the Supplementary Table S1.

NR treatment increased intracellular NAD^+^ levels in vitro in both murine (H5V) and human (HMEC-1) microvascular endothelial cell lines compared to the controls. This effect in H5V and HMEC-1 cells was significantly suppressed by ENT1 inhibition, accompanied by a decrease in intracellular NR. Combining NR with a PNP inhibitor did not result in a further increase in NAD^+^, but nicotinamide riboside accumulation was significantly higher when PNP activity was blocked (Figure 1a,c). Besides the slight decrease in the ATP/ADP ratio and adenylate energy charge (AEC) in HMEC-1 following incubation with NR and PNPi combined, the total adenine nucleotide pool (TAN), ATPD/ADP and NAD^+^/NADH after incubation with NR were preserved in both tested cell lines (Figure 1b,d–f).

### 2.2. NR Does Not Affect Mitochondrial Respiration or Glycolysis in HMEC-1 Cells After 24 h Incubation

**Figure 2:**
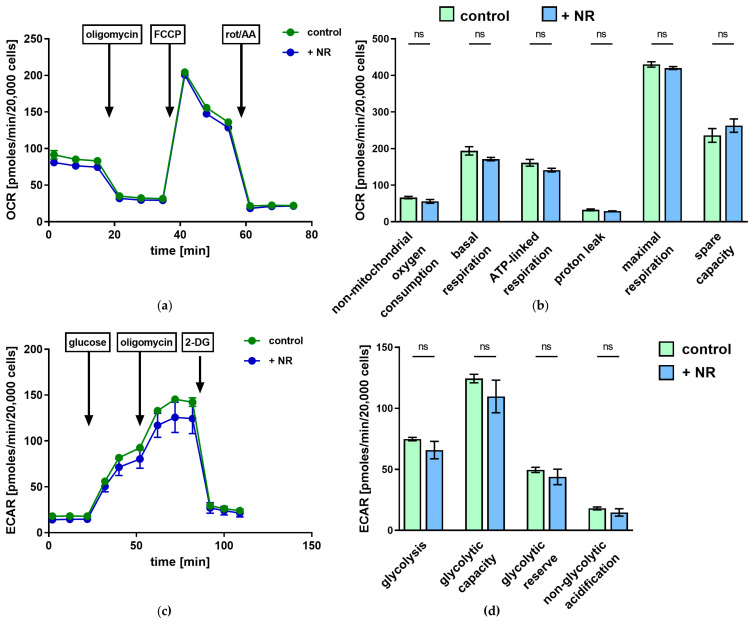
Oxygen consumption rate (OCR) and extracellular acidification rate (ECAR) in control and NR-treated HMEC-1 cells measured with Seahorse XF Analyzer. (**a**) OCR traces obtained during the mitochondrial stress test following sequential injections of oligomycin, FCCP, and rotenone/antimycin A (rot/AA), (**b**) quantification of parameters derived from OCR, (**c**) ECAR traces obtained during the glycolysis stress test following sequential injections of glucose, oligomycin, and 2-deoxy-D-glucose (2-DG), and (**d**) quantification of parameters derived from ECAR. Arrows indicate the time points of compound injections. Results are presented as mean ± SEM, *n* = 3, ns—*p* ≥ 0.05, multiple unpaired two-tailed Student’s *t*-tests with Holm–Sidak correction for multiple comparisons. Exact *p* values are provided in the Supplementary Table S1.

The effects of NR supplementation on the energy metabolism of human endothelial cells were further investigated using oxygen consumption rate (OCR) and extracellular acidification rate (ECAR) measurements with Seahorse extracellular flux analysis. We did not observe changes in OCR and ECAR measured over time between NR-treated and control cells (Figure 2a,c). There were no statistically significant differences in quantification of non-mitochondrial oxygen consumption, basal respiration, ATP-linked respiration, proton leak, maximal respiration, and spare respiratory capacity derived from OCR (Figure 2b), and glycolysis, glycolytic capacity, glycolytic reserve, and non-glycolytic acidification derived from ECAR (Figure 2d).

### 2.3. Nicotinamide Riboside Alters Extracellular Adenine Nucleotide Catabolism in Endothelial Cells and WT Mice Aortas

**Figure 3:**
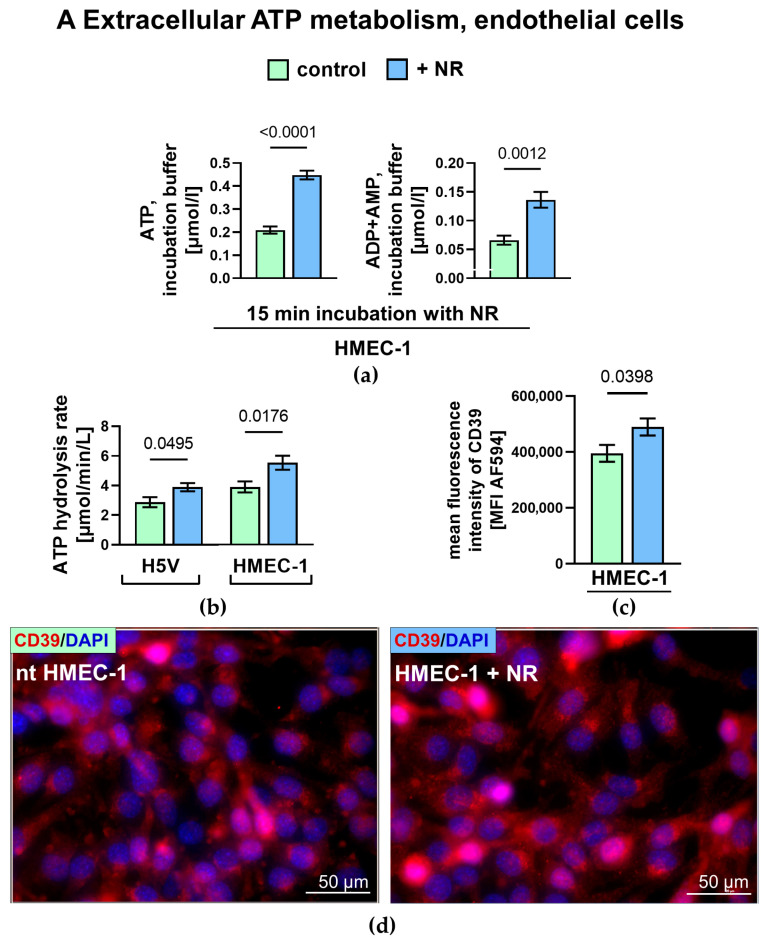

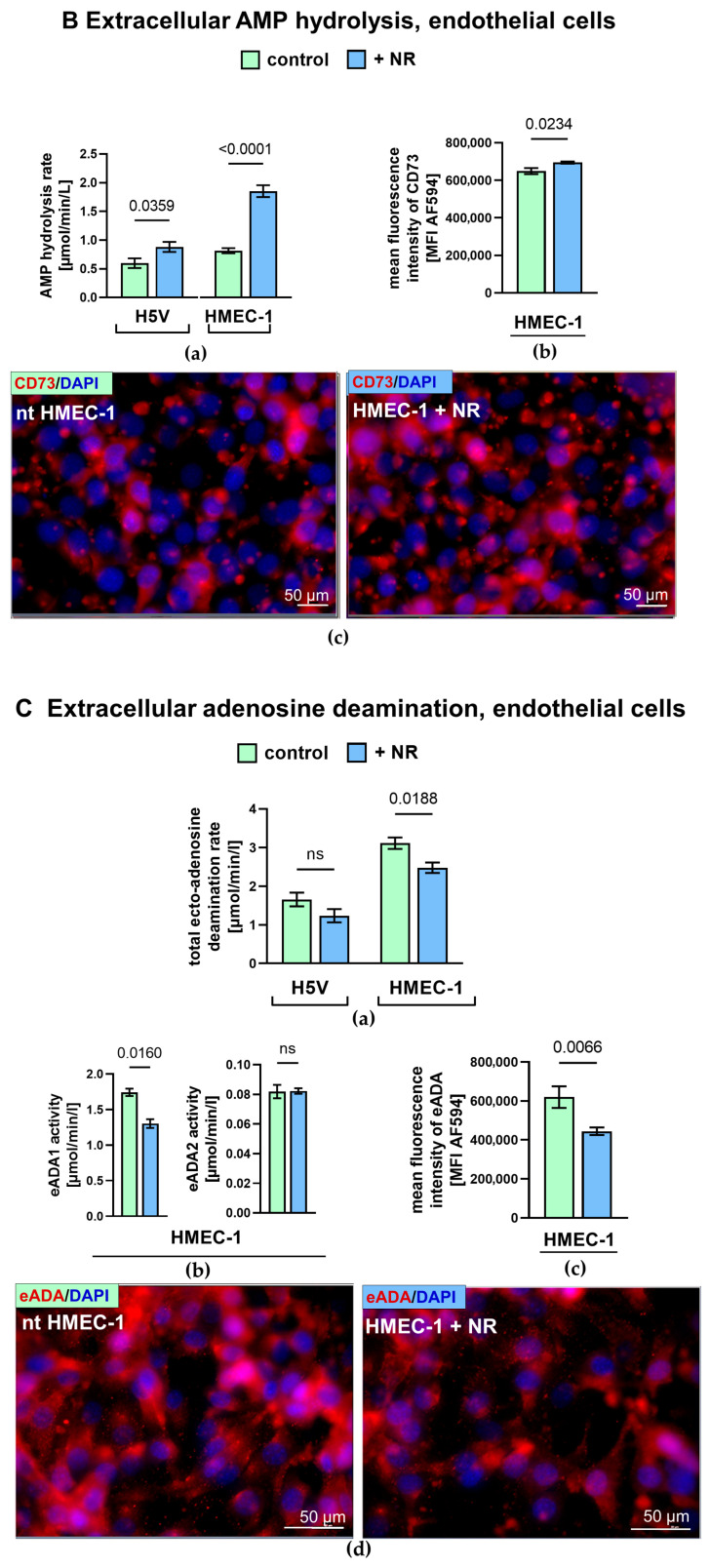

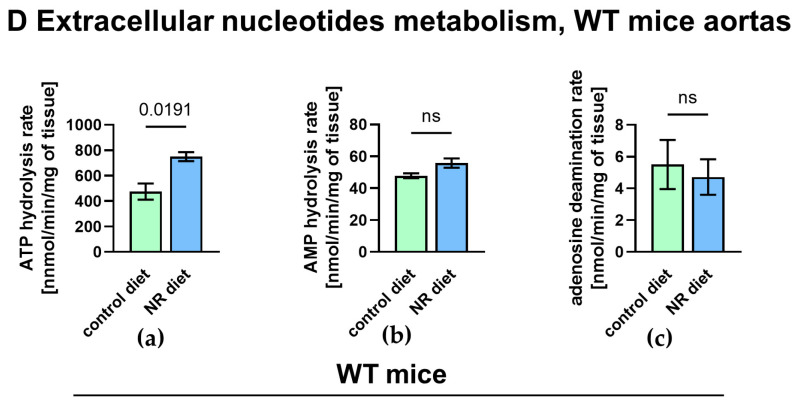
(**A**) Extracellular ATP metabolism in endothelial cells. (**a**) Concentrations of adenine nucleotides in medium after 15 min incubation of HMEC-1 cells with NR, (**b**) extracellular ATP hydrolysis rate in H5V and HMEC-1 cells (**c**), quantitative analysis and (**d**) representative images of immunofluorescence staining of CD39 protein in HMEC-1 cells. Results are presented as mean ± SEM, *n* = 4, (**a**) 5–8, (**b**) 10–12, (**c**) Student’s *t*-test. (**B**) Extracellular AMP metabolism in endothelial cells. (**a**) AMP hydrolysis rate in H5V and HMEC-1 cells, (**b**) quantitative analysis and (**c**) representative images of immunofluorescence staining of CD73 protein in HMEC-1 cells. Results are presented as mean ± SEM, *n* = 5–8, (**a**) 9, (**b**) Student’s *t*-test. (**C**) Extracellular adenosine deamination in endothelial cells. (**a**) Total ecto-adenosine deamination rate in H5V and HMEC-1 cells, (**b**) eADA1 and eADA2 activity in HMEC-1 cells, (**c**) quantitative analysis and (**d**) representative images of immunofluorescence staining of eADA protein in HMEC-1 cells. Results are presented as mean ± SEM, *n* = 5–8, (**a**,**b**) 15, (**c**) Student’s *t*-test. Examples of fields of view were imaged with an AxioObserver 7 fluorescence microscope at 40× magnification, ZEN 3.3 software. DAPI (blue)– nuclei; Alexa Fluor 594 (red)–CD39, CD73 or eADA protein. Data in (**A**(**c**),**B**(**b**),**C**(**c**)) were obtained from three independent biological replicates per group with 3–5 fields of view. (**D**) Extracellular nucleotides metabolism on the surface of WT control and NR-supplemented mice aortas. (**a**) ATP hydrolysis, (**b**) AMP hydrolysis and (**c**) adenosine deamination rate. Results are presented as mean ± SEM, *n* = 3, Student’s *t*-test. Exact *p*-values for statistically significant comparisons are shown on the graphs; non-significant differences are marked as ns (*p* ≥ 0.05).

To evaluate the effect of NR stimulation on the extracellular enzyme activity of endothelial cells, we performed the quantification of extracellular ATP levels and its degradation products during incubation with NR, as well as the ecto-enzymes assay as previously described [24]. Within the first minutes following the addition of NR, we observed significantly higher levels of extracellular ATP, ADP, and AMP in the incubation medium compared to the control cell medium. Moreover, 24 h incubation with NR significantly enhanced ATP hydrolysis on the surface of HMEC-1 and H5V cells (Figure 3A). We also observed an increased AMP hydrolysis rate in NR-treated endothelial cells (Figure 3B). The predominant role of CD73 in AMP hydrolysis at the endothelial surface was confirmed by the addition of its inhibitor (AOPCP), which markedly inhibited extracellular AMP hydrolysis (Supplementary Figure S1). Additionally, nicotinamide riboside supplementation decreased the total extracellular adenosine deamination rate in human ECs. Despite the slight downward trend, we did not observe a statistically significant change in adenosine deamination on the surface of murine ECs. The reduced total eADA activity in HMEC-1 cells resulted from attenuated eADA1 activity, as no statistically significant differences were observed in the eADA2 isoenzyme rate (Figure 3C). Consistently, immunofluorescent staining showed an increased quantity of CD39 and CD73 proteins and a reduction in eADA quantity in nicotinamide riboside-treated HMEC-1 cells compared to the control. To validate these in vitro findings, we assessed ecto-enzyme activities on the surfaces of isolated aortas from wild-type mice (C57BL/6J) that had been supplemented with nicotinamide riboside for 12 weeks. ATP hydrolysis at the aortic surface was markedly increased following NR supplementation, and an increasing trend was observed for AMP hydrolysis. In contrast, no changes were detected in adenosine deamination rate.

### 2.4. Extracellular NAD^+^ Hydrolysis in HMEC-1 Cells

**Figure 4:**
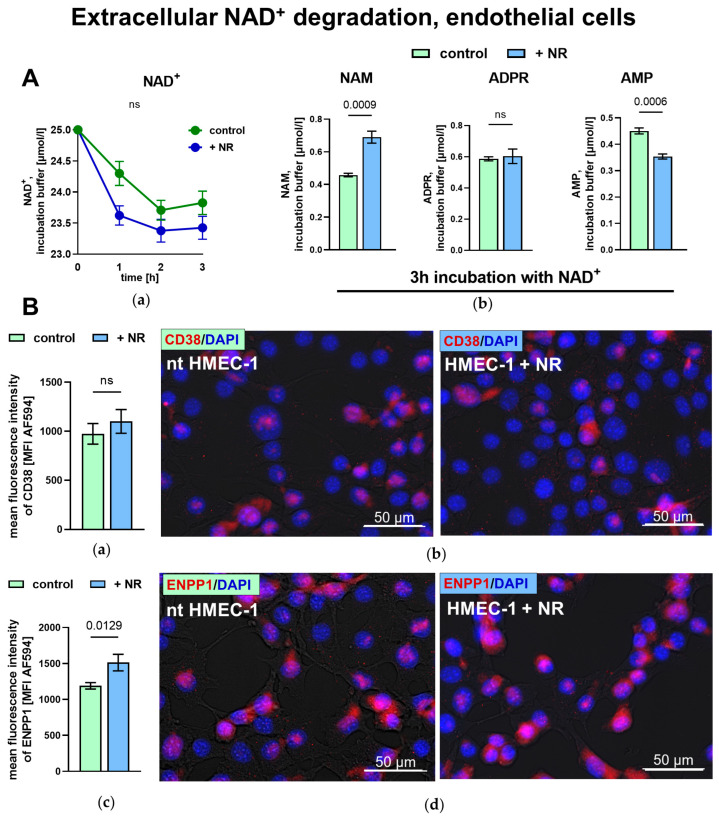
Extracellular NAD^+^ metabolism in NR-treated human endothelial cells. (**A**) (**a**) Extracellular NAD^+^ degradation in time and (**b**) concentrations of NAM, ADPR and AMP after 3 h incubation of HMEC-1 cells with NAD^+^. Results are presented as mean ± SEM, *n* = 4, two-way mixed-effects model, Tukey’s post hoc (**a**) or Student’s *t*-test (**b**). (**B**) Quantitative analysis and representative images of immunofluorescence staining of (**a**,**b**) CD38 and (**c**,**d**) ENPP1 protein in HMEC-1 cells. Results are presented as mean ± SEM, *n* = 6–8, Student’s *t*-test. Examples of fields of view were imaged with an AxioObserver 7 fluorescence microscope at 40× magnification, ZEN 3.3 software. DAPI (blue)–nuclei; AF594 (red)–CD38 or ENPP1 protein. Results in (**B**) were obtained from two biological replicates per group, with 3–4 fields of view per well. Exact *p*-values for statistically significant comparisons are shown on the graphs; non-significant differences are marked as ns (*p* ≥ 0.05).

In addition to ATP-derived purine metabolites, extracellular NAD^+^ degradation may represent an additional source of AMP in endothelial cells. Both control and NR-treated HMEC-1 cells exhibited a time-dependent degradation of exogenous NAD^+^, and the products of extracellular NAD^+^ degradation were detected in the incubation medium. Following NR treatment, extracellular NAM levels were significantly increased, ADPR remained unchanged, while AMP levels were significantly reduced compared to the control (Figure 4A). Immunofluorescent staining of CD38 revealed a low but detectable level of this protein in both the control and NR-treated HMEC-1 cells, with no significant differences between groups, while ENPP1 staining showed that NR treatment increased ENPP1 levels compared to the control (Figure 4B).

We also investigated whether sirtuin signaling contributes to the observed extracellular effects of NR by assessing the expression of SIRT1, SIRT2, and SIRT3 in murine endothelial cells. No significant differences were observed between the control and NR-treated cells (Supplementary Figure S2), suggesting that under our experimental conditions, NR did not affect sirtuin expression.

### 2.5. Nicotinamide Riboside Attenuates Adhesion of Circulating Immune Cells and Platelets to HMEC-1 Cells

**Figure 5:**
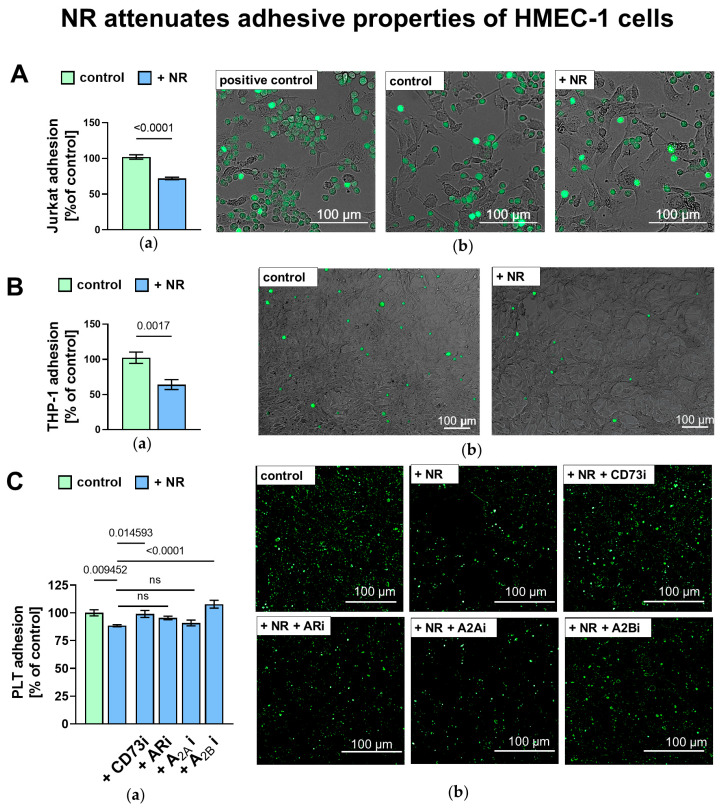
(**A**) Jurkat cell adhesion to HMEC-1 cells. (**a**) Quantitative analysis and (**b**) representative images. Results are expressed as percentage of mean fluorescence intensity (MFI) relative to the non-treated control. NF-α-stimulated cells served as a positive control for adhesion and were excluded from statistical analysis. Representative images show 4% formalin-fixed wells with 5-carboxyfluorescein-labeled Jurkat cells (green) and unlabeled HMEC-1 cells. (**B**) THP-1 cell adhesion to HMEC-1 cells. (**a**) quantitative analysis and (**b**) representative images. Results are expressed as percentage of MFI relative to the non-treated control. Representative images show 4% formalin-fixed wells with 5-carboxyfluorescein-labeled THP-1 cells (green) and unlabeled HMEC-1 cells. (**C**) Platelet adhesion to HMEC-1 cells. (**a**) Quantitative analysis and (**b**) representative images of activated platelet adhesion to non-treated HMEC-1 or NR-treated cells in the presence of CD73 inhibitor and/or adenosine receptor antagonists, expressed as percentage of MFI relative to control. CD73i—AOPCP; ARi—non-selective adenosine receptor inhibitor DPSPX; A2Ai—A2A receptor antagonist istradefylline; A2Bi—A2B receptor antagonist PSB1115. Representative images show 1.1% formalin-fixed wells with CD31/CD61-labeled platelets (green). Data were obtained from three biological replicates per group, with 2–3 fields of view per well per experiment. Examples of fields of view were imaged with an AxioObserver 7 fluorescence microscope with Nomarski contrast at 10× or 20× magnification, ZEN 3.3 software. Results are presented as mean ± SEM, *n* = 6–9, Student’s *t*-test (**A**,**B**) or one-way ANOVA followed by Dunnett’s post hoc test (**C**). Exact *p*-values for statistically significant comparisons are shown on the graphs; non-significant differences are marked as ns (*p* ≥ 0.05).

To investigate whether nicotinamide riboside affects cell adhesion to the endothelial monolayer, NR-pretreated HMEC-1 cells were co-incubated with immune cells and platelets. Adhesion of Jurkat-line T cells and THP-1 monocytes/macrophages to the NR-treated endothelial monolayer was significantly reduced compared to the control (Figure 5A,B). The addition of exogenous AMP further decreased the Jurkat cells’ adhesion, while inhibition of CD73 activity reversed this anti-adhesive effect (Supplementary Data). A similar pattern was observed in the adhesion of activated platelets to HMEC-1 cells pretreated with NR, CD73 inhibitor and adenosine receptor (AR) antagonists. Platelet adhesion to NR-treated endothelial cells was attenuated compared to the control but abolished when CD73 activity on the surface of ECs was inhibited. Moreover, we did not observe a decrease in platelet adhesion to NR-treated HMEC-1 cells when the A2B AR antagonist was present. (Figure 5C).

## 3. Discussion

Replenishment of the intracellular NAD^+^ pool has emerged as a promising therapeutic strategy for various pathological conditions, including metabolic, neurodegenerative, and cardiovascular diseases, as well as age-related functional decline. In all these pathologies, we observe depletion of cellular NAD^+^ as well as changes in its metabolism [3,4]. As the NAD^+^ plays a key role in maintaining cellular bioenergetics, DNA repair, and stress response, it is suggested that restoring its intracellular levels can improve the cell defense mechanism against metabolic and oxidative stress [25]. Among the precursors of NAD^+^, nicotinamide riboside is currently regarded as the most effective and safe option for enhancing intracellular NAD^+^ levels. Unlike other metabolites such as nicotinic acid or nicotinamide, NR exhibits favorable pharmacokinetics, causes minimal adverse effects even in high doses, and has been recognized as safe for human consumption [8,26,27]. In this study, we demonstrated that NR is metabolized intracellularly and significantly enhances NAD^+^ levels in murine and endothelial cells, as well as in murine aortic tissue ex vivo. NR is intracellularly converted to NAD^+^ via a two-step pathway involving nicotinamide riboside kinases (NRK1/2) and nicotinamide mononucleotide adenylyltransferase (NMNAT); however, NR can also be metabolized by purine nucleoside phosphorylase (PNP), potentially limiting its availability for NAD^+^ biosynthesis [28,29]. Our results suggest that purine nucleoside phosphorylase acts on NR intracellularly, but this process has no effect on NAD^+^ increase following treatment in both murine and human ECs. This confirms that NR can be an effective precursor for NAD^+^ biosynthesis in endothelial cells in physiological conditions. However, it should be noted that systemic effects of metabolizing NR by PNP can differ from endothelial in vitro models, as PNP expression and functionality is more prominent in other types of cells, especially immune cells. In this study we also show that NR supplementation had no effect on intracellular adenine nucleotide concentrations. Consistently, extracellular flux analysis revealed no significant changes in oxygen consumption rate (OCR) or extracellular acidification rate (ECAR), indicating that NR treatment has no effect on cellular energy metabolism in HMEC-1 cells and supporting the metabolic safety of NR. However, these observations should be interpreted with caution, given the relatively small sample size and the duration of NR exposure, which may not fully reflect the effects in longer-term supplementation. In physiological conditions, endothelial cells rely mainly on glycolysis for sustaining energy status [30]. As previously mentioned, several studies have reported that NR improves mitochondrial biogenesis, respiration, and stress resistance, especially in metabolically active tissues. However, data on NR’s effects on glycolysis and mitochondrial function in physiological endothelial cells remain limited, and our study is one of the first to address this. Future studies require more functional tests that would complement current observations.

The effects of NR supplementation on extracellular adenine nucleotide catabolism in HMEC-1 cells are an important aspect of our studies due to the key role these enzymes play in regulating vascular homeostasis and purinergic signaling. Endothelial cells express ecto-enzymes such as CD39, CD73, and eADA, which not only modulate extracellular levels of nucleotides but also affect immune cell recruitment, inflammation and thrombosis [31,32]. One of the main ecto-enzymes in the context of NAD^+^ metabolism is NAD^+^ nucleosidase (CD38), which metabolizes NAD^+^ and NMN to ADPR and cADPR [33]. ADPR can then be metabolized to AMP by ecto-nucleotide pyrophosphatase/phosphodiesterase 1 (ENPP1, CD203a) and AMP to adenosine by CD73 [34]. There are reports indicating that HMEC-1 cells do not exhibit CD38 hydrolase activity [35]. However, our results demonstrate the presence of the CD38 and ENPP1 proteins in HMEC-1 cells, and a low but detectable level of extracellular NAD^+^ hydrolysis at the surface of HMEC-1 cells, leading to the formation of measurable products. Additionally, we show that NR supplementation affects extracellular NAD^+^ metabolism, as reflected by altered levels of NAM and AMP following the degradation of exogenous NAD. The decreased extracellular AMP levels despite increased ENPP1 expression suggest enhanced metabolic flux through this pathway, with rapid conversion of AMP to downstream metabolites, likely mediated by increased CD73 activity. In this study, we demonstrated an increased quantity of CD39 and CD73 proteins in HMEC-1 cells, as well as an increased ATP and AMP hydrolysis rate on both murine and human EC surfaces. These observations are partially supported by preliminary ex vivo data obtained from aortic tissue of NR-supplemented mice, which showed increased ATP hydrolysis, while AMP hydrolysis and adenosine deamination remained unchanged. Given the limited sample size, these findings should be interpreted cautiously. AMP hydrolysis in the presence of CD73 inhibitor AOPCP was almost completely inhibited, which confirmed CD73 as the main enzyme involved in extracellular AMP catabolism in HMEC-1. However, the observed changes in ecto-enzyme protein levels based on immunofluorescence should be interpreted with caution, as this approach does not provide definitive quantitative evidence of protein expression, so our findings are supported primarily by the functional readouts of ATP and AMP hydrolysis rates. In physiological conditions, extracellular nucleotide concentrations are maintained at low basal levels, and even small local increases in ATP are rapidly sensed and metabolized by CD39 to AMP and then by CD73 to adenosine [36]. Additionally, extracellular ATP can also be directly hydrolyzed to AMP by ENPP1, bypassing the CD39-mediated pathway [37]. Classically, increased extracellular ATP release is considered a consequence of cellular damage, where ATP is released upon membrane disruption or cell stress. However, there is a rising amount of evidence indicating that ATP is also released under physiological conditions through regulated mechanisms [38]. One of those mechanisms described for human endothelial cells is the expression of pannexin-1 (Panx1) channels, as their inhibition effectively suppressed ATP release from stimulated HUVEC cells [39]. Our study showed that the incubation of HMEC-1 cells with NR led to increased ATP release and elevated levels of ADP and AMP in medium; however, we were unable to reliably quantify extracellular adenosine levels in a nucleotide release assay as its rapid turnover and low concentrations makes the detection challenging, particularly in the absence of exogenous substrate stimulation [40]. Increased ATP release in physiological HMEC-1 cells may be a secondary effect of boosting intracellular NAD^+^ levels, as a similar mechanism was observed after NMN supplementation in cancer cells [41]. Collectively, these findings indicate that NR promotes a coordinated shift in extracellular nucleotide metabolism, characterized by increased ATP-degrading ecto-enzymes and CD73 activity, as well as reduced eADA-mediated degradation, which favors the accumulation of adenosine. Elevated adenosine concentrations, resulting from CD39/ENPP1-CD73 activity and reduced eADA-mediated degradation, would represent a favorable phenotype of ECs in the context of preventing endothelial dysfunction. By acting on A2A and A2B receptors, adenosine can inhibit the expression of adhesion molecules on endothelial cells, suppressing leukocyte adhesion and platelet infiltration [42]. It was reported that endothelial cell lines exhibit differences in the expression of adenosine receptors, with HMEC-1 cells preferentially expressing A2B receptors [43]. In our study, we showed that NR supplementation of HMEC-1 reduced the adhesion of Jurkat cells and platelets, while inhibition of CD73 attenuated these effects. Moreover, inhibition of HMEC-1 A2B receptors together with NR treatment also resulted in the reversal of the anti-adhesive effect. In contrast, no such effect was observed upon a non-selective blockade of adenosine receptors, which may reflect compensatory or opposing roles of individual receptor subtypes, by masking the contribution of A2B signaling. These findings further support the notion that NR supplementation modulates extracellular enzyme activity, leading to increased adenosine availability, and that the anti-adhesive effects of NR are adenosine-dependent. We also observed the attenuated adhesion of THP-1 cells to the NR-treated HMEC-1 monolayer, which is consistent with a shift in extracellular purine metabolism toward adenosine generation and an anti-adhesive endothelial phenotype. Direct reports indicate that extracellular adenosine inhibits the TNF-α-induced expression of E-selectin, ICAM-1, and VCAM-1, resulting in reduced THP-1 adhesion to endothelium in vitro. However, they also suggest that these anti-inflammatory effects may involve pathways other than the direct activation of adenosine receptors [44].

Although the discussion above outlines potential mechanisms through which nicotinamide riboside can affect extra-enzymatic activity, the specific metabolic pathways underlying these effects after NR supplementation are yet to be reported. Interestingly, there are existing reports showing that dietary NR supplementation increases ATP and ADP, as well as adenosine content in human peripheral blood mononuclear cells [45]. However, to our knowledge, this study is among the first to focus on NR effects on adenosine pathways at the cellular level. Our findings are particularly relevant in the context of early endothelial dysfunction in atherosclerosis, where impaired adenosine signaling can exacerbate vascular inflammation and plaque formation. However, it is important to note that the effects observed in our study were demonstrated in endothelial cells under physiological, non-inflammatory conditions. It remains undetermined whether similar responses to NR stimulation would be maintained in endothelial cells already exposed to pathological stressors, as well as how intercellular interactions between different cell types within the vascular wall in vivo can modulate these extracellular pathways. While our results suggest that NR supplementation may support endothelial homeostasis and help prevent early dysfunction, further studies are required to fully assess its biological and clinical effects. In particular, the current state of knowledge does not allow the reliable prediction of NR outcomes in heterogeneous patient populations using over-the-counter supplements. Overall, these findings provide new insight into the role of NAD^+^ precursors in regulating extracellular purine metabolism and endothelial function, highlighting a previously underappreciated mechanism linking NAD^+^ metabolism with vascular homeostasis.

## 4. Materials and Methods

### 4.1. In Vitro Cell Culture

Murine-immortalized heart endothelial cell line (H5V) was kindly gifted by Dr. Habil. Patrycja Koszalka, from the Department of Cell Biology, Faculty of Medical Biotechnology, Medical University of Gdansk, Poland. Human microvascular endothelial cell line (HMEC-1), human T lymphoblast line (Jurkat, Clone E6-1), and human monocyte cell line (THP-1) were obtained from ATCC (Manassas, VA, USA). HMEC-1 cells were cultured in MCDB131 medium with 10% FBS, 10 ng/mL epidermal growth factor, 1 μg/mL hydrocortisone, 10 mM L-glutamine, and 1% penicillin/streptomycin, H5V in high-glucose DMEM with 10% FBS, 2 mM L-glutamine, and 1% penicillin/streptomycin, and Jurkat and THP-1 cells in RPMI 1640 medium with 10% FBS and 1% penicillin/streptomycin. All cell cultures were maintained at 37 °C, 5% CO_2_. All experiments were conducted between the third and sixth cell passages.

### 4.2. Measurement of Intracellular Nucleotide Concentration

To assess intracellular NAD^+^, NADH, ATP, ADP, and AMP levels in the HMEC-1 cell line, cells were seeded in 24-well culture plates (Corning, Corning, NY, USA) at 0.6 × 105 density in 1 mL of complete growth medium. After reaching confluence, cell monolayers were washed twice with PBS and treated with 500 µM nicotinamide riboside chloride (Niagen, Los Angeles, CA, USA) and/or 10 µM S-(4-Nitrobenzyl)-6-thioinosine (NBTI) and 1.5 µM purine nucleoside phosphorylase (PNP) inhibitor forodesine (Cayman Chemical, Ann Arbor, MI, USA) in growth medium w/o FBS. After 24 h of incubation, the medium was collected, and the cells were rinsed three times with PBS. Next, 300 μL of ice-cold 0.4 M HClO_4_ was added, and the samples were immediately frozen at −80 °C. After that, the samples were thawed on ice and then frozen again at −80 °C. Following the second thawing on ice, samples were collected and neutralized with 3 M K_3_PO_4_ to a pH of 6–7, then centrifuged at 22,000× *g* and 4 °C for 10 min. The supernatants were then analyzed for intracellular nucleotide concentrations using an RP-HPLC method, as described by us in previous work [31]. Results were expressed as nmol/mg of protein.

### 4.3. Extracellular ATP Release and Ecto-Enzymes Activity Assay

To assess extracellular ATP release in HMEC-1 cells, confluent HMEC-1 cells in 24-well culture plates were washed twice with PBS. Then, 1 mL of HBSS (Corning, Corning, NY, USA) with NR at a final concentration of 500 µM was added. Sample collection was initiated immediately after the addition of the incubation medium. Samples were collected at 0, 1, 5, 10, and 15 min time points and immediately snap-frozen on dry ice to prevent nucleotide degradation. Concentrations of extracellular nucleotides were subsequently determined using high-performance liquid chromatography (HPLC). Results are expressed as µmol/L. To determine cell–surface enzyme activity, confluent H5V or HMEC-1 cells in 24-well culture plates were washed twice with PBS and incubated in 1 mL of HBSS (Corning, Corning, NY, USA) at 37 °C with substrates ATP, AMP, or adenosine at a final concentration of 50 µM. Samples were collected at 0, 15, and 30 min time points and analyzed using RP-HPLC as described above. ATP hydrolysis rate and eADA2 activity were assessed in the presence of 5mM eADA1 inhibitor erythro-9-(2-hydroxy-3-nonyl)adenine (EHNA; Merck KGaA, Darmstadt, Germany), and AMP hydrolysis rate in the presence of 5 mM EHNA and 50 µM CD73 inhibitor adenosine 5′-(α,β-methylene)diphosphate sodium salt (AOPCP; Merck KGaA, Darmstadt, Germany). eADA1 activity was calculated by subtracting the eADA2 activity from the total eADA activity. The results were expressed as the product increase over time (µmol/min/L). For the extracellular NAD^+^ degradation assay, cells were incubated with a NAD^+^ final concentration of 25 µM. At defined time points (30, 60, 120, 180, and 240 min), medium samples were collected and analyzed to determine the concentrations of NAD^+^, NAM, AMP, and ADPR. Results were expressed as endpoint concentrations in medium for NAM, AMP and ADPR and as time-course of changes in NAD^+^ concentrations in medium (µmol/L).

### 4.4. Animal Maintanance and Assesment of Extracellular Activity on the Surface of WT Mice Aortas

All experiments on animals were conducted following a Guide for the Care and Use of Laboratory Animals by the European Parliament (Directive 2010/63/EU), and were approved by the Local Ethical Committee for Animal Experimentation (21/2025). Briefly, 12-week-old male wild-type C57BL/6J mice were fed a regular chow diet or nicotinamide riboside-supplemented (Niagen, Los Angeles, CA, USA) diet at a dose of 500 mg of NR/kg body weight. After 12 weeks, mice were anesthetized with a mixture of ketamine/xylazine. Isolated aortas were carefully rinsed in PBS and the surrounding connective and adipose tissue were thoroughly cleaned. The aorta was then sectioned and opened longitudinally along the ventral wall. Aortic segments were incubated in 1 mL of HBSS (Corning, Corning, NY, USA) at 37 °C and the ecto-enzyme activity assay was performed as described in Section 4.4. Following the assay, the aortic fragments were dried and weighed. Enzymatic activities were expressed as µmol/min/mg of dry tissue.

### 4.5. Immunofluorescence Staining

HMEC-1 cells were seeded onto 96-well optical plates (Thermo Fisher Scientific Inc., Waltham, MA, USA) at a density of 0.1 × 105. After 80% confluence was reached, cells were incubated with 500 µM nicotinamide riboside in serum-free medium for 24 h. Then, the cells were washed three times with PBS and fixed with a 4% formaldehyde solution for 15 min at 37 °C. Nonspecific antibody binding was blocked by incubating the fixed cells with a 10% goat serum and 1% BSA solution in PBS for 1 h. Next, cells were washed and incubated with PBS-diluted antibody solutions: CD39/ENTPD1 polyclonal antibody (14211-1-AP, Proteintech, Rosemont, IL, USA, 1:100), 5′-Nucleotidase/CD73 Antibody (NBP-185740, Novus Biologicals™, Centennial, CO, USA, 1:100), ADA polyclonal antibody (13328-1-AP, Proteintech, Rosemont, IL, USA, 1:100), CD38 Antibody (1G7F4) (NBP2-25250, Novus Biologicals™, Centennial, CO, USA, 1:100), and ENPP1 policlonal antibody (NBP2-38945, Novus Biologicals™, Centennial, CO, USA, 1:100) for 1 h. Cells were then rinsed and incubated for 30 min with secondary antibodies: Alexa Fluor^®^ 594 AffiniPure Goat Anti-Mouse IgG (115-585-003, Jackson ImmunoResearch Europe Ltd., Ely, UK, 1:600) for CD39, eADA, and CD38, or Alexa Fluor^®^ 594 AffiniPure Goat Anti-Rabbit IgG (111-585-003, Jackson ImmunoResearch Europe Ltd., Ely, UK, 1:600) for CD73 and ENPP-1. Finally, cell nuclei were counterstained with DAPI (MBD0015, Merck KGaA, Darmstadt, Germany, 1:1000) for 5 min. Stainings were imaged using the AxioObserver 7 fluorescence microscope and ZEN 3.3 software (Carl Zeiss Inc., Dresden, Germany). Protein quantity was expressed as mean fluorescence intensity (MFI) for 594 nm.

### 4.6. Immune Cells Adhesion Assay

HMEC-1 cells were seeded onto 96-well optical plates (Merck Millipore, Darmstadt, Germany). After reaching confluence, cells were incubated with 500 µM NR or 1 ng/mL TNFα (positive control) for 24 h, with 500 µM NR and 50 µM AOPCP for 24 h, or 50 µM AMP for the final 30 min. Then, the cell monolayer was washed twice with PBS, and labeled Jurkat or THP-1 cells were added at 1.25 × 104 in 50 µL of serum-free medium. Jurkat and THP-1 cells were labeled with 5-carboxyfluorescin succinimidyl ester (CFSE; 21888, Merck KGaA, Darmstadt, Germany) directly before adding to the endothelial monolayer as described previously [32]. Cells were co-incubated at 37 °C, 5% CO_2_ for 30 min. After co-incubation, non-adhered immune cells were removed by three rinses with PBS, and wells were fixed with ice-cold methanol for 5 min. Adhesion to the HMEC-1 monolayer was measured as the area of residual green fluorescence staining using an AxioObserver 7 microscope and ZEN 3.3 software (Carl Zeiss Inc., Dresden, Germany), and expressed as a percentage of adhesion for control cells.

### 4.7. Human Platelets Isolation and Platelets Adhesion Assay

Whole blood from a healthy volunteer was collected into a 3.2% sodium citrate BD Vacutainer^®^ tube (Becton, Dickinson and Company, Franklin Lakes, NJ, USA) and centrifuged immediately at 190× *g*, room temperature for 15 min. Then, platelet-rich plasma (PRP) was carefully collected using a wide-tip pipette, transferred to a new tube, and centrifuged at 2500× *g* at room temperature for 5 min. After the second centrifugation, platelet-poor plasma (PPP) was discarded, and the pellet was gently suspended in 1 mL of HBSS w/o Ca^2+^ and Mg^2+^ (Corning, Corning, NY, USA). The platelet suspension was left for 15 min at room temperature before proceeding to the next step.

Isolated human platelets were activated by a 5 min incubation with 5 µM ADP at 37 °C while being continuously shaken. Then, the platelets were centrifuged at 190× *g*, 21 °C, for 2 min, and subsequently resuspended in PBS to a final concentration of 2 × 10^7^/mL. Platelets were then labeled with the anti-CD41/CD61 mouse antibody (SAB4700792, Merck, Darmstadt, Germany, 1:100) for 1 h. After incubation, the platelets were rinsed three times with PBS and centrifuged at 190× *g*, 21 °C, for 3 min. Then, platelets were incubated for 30 min with a secondary Alexa Fluor^®^ 488 AffiniPure Goat Anti-Mouse IgG (115-545-003, Jackson ImmunoResearch Europe Ltd., Ely, UK, 1:600), rinsed three times with PBS, centrifuged at 190× *g*, 21 °C, for 3 min, and resuspended in HBSS.

HMEC-1 cells were seeded onto 96-well optical plates (Merck Millipore, Darmstadt, Germany) and treated for 24 h with 500 µM NR or 500 µM NR and 50 µM AOPCP, DPSPX, istradefylline or PSB1115. Labeled platelets were added at a final concentration of 70 µL to HMEC-1 monolayers and incubated for 30 min at 37 °C. After co-incubation, non-adhered platelets were removed by washing three times with PBS, and the remaining cells were fixed for 30 min with 1.1% formalin-PBS solution. Activated platelets’ adhesion to the HMEC-1 monolayer was quantified as mean fluorescence intensity of Alexa Fluor 488-labeled CD41/CD61 using an AxioObserver 7 microscope and ZEN 3.3 software (Carl Zeiss Inc., Dresden, Germany).

### 4.8. Seahorse Extracellular Flux Analysis

HMEC-1 cells were initially seeded at a concentration of 20,000 cells per well in an Agilent Seahorse microplate (Agilent, Santa Clara, CA, USA) and allowed to grow until they reached 80% confluency (80 µL of culture medium per well, maintained at 37 °C with 5% CO_2_). The cells were then treated with 500 µM NR for a duration of 24 h. For the cell mito stress test, the medium was replaced 45 min prior to the XFp assay with a medium containing 10 mM glucose, 2 mM glutamine, and 1 mM pyruvate, and the cells were incubated in an environment without CO_2_. During the assay, 1.5 µM oligomycin, 1 µM carbonyl cyanide 4-(trifluoromethoxy) phenylhydrazone (FCCP), and 0.5 µM rotenone with antimycin were added in sequence. Before conducting the glycolysis stress test, the medium was switched to XFp assay medium with 2 mM glutamine (Agilent, Santa Clara, CA, USA). For the analysis, 10 mM glucose, 1.0 µM oligomycin, and 50 µM 2-DG were sequentially introduced. The Agilent Seahorse XFp Analyzer (Agilent, Santa Clara, CA, USA) was used to carry out the analysis as described earlier.

### 4.9. Statistical Analysis

Statistical analysis was performed using Graph Pad Prism version 9.0.0 (Intuitive Software for Science, San Diego, CA, USA). All data were first tested for normality. Comparisons between two groups were conducted using an unpaired two-tailed Student’s *t*-test. For more than two groups, a one-way ANOVA was applied, followed by multiple-comparison post hoc tests: Dunnett’s, Tukey’s, or Holm–Sidak’s. Where appropriate, a two-way ANOVA with a mixed-effects model and multiple unpaired two-tailed Student’s *t*-tests with Holm–Sidak correction for multiple comparisons were applied. Results are presented as mean ± standard mean error (SEM) unless stated otherwise. A *p*-value < 0.05 was considered statistically significant.

## 5. Conclusions

Nicotinamide riboside supplementation increases intracellular NAD^+^ levels in endothelial cells without affecting cellular energy metabolism, while significantly modulating extracellular purine metabolism. NR promotes a coordinated shift toward enhanced adenosine generation through increased CD39/CD73 activity and reduced eADA-mediated degradation. This metabolic reprogramming is associated with reduced immune cell and platelet adhesion, suggesting a potential anti-inflammatory and vasoprotective mechanism. These findings reveal a link between NAD^+^ metabolism and extracellular purinergic signaling with potential relevance for vascular homeostasis and early endothelial dysfunction.

## Data Availability

The raw data supporting the conclusions of this article will be made available by the authors on request.

## Supplementary Materials

The following supporting information can be downloaded at: https://www.mdpi.com/article/10.3390/ijms27073267/s1.

## Abbreviations

The following abbreviations are used in this manuscript:

ADO	Adenosine
ADP	Adenosine diphosphate
AEC	Adenylate energy charge
AMP	Adenosine monophosphate
AOPCP	5′-(α,β-methylene)diphosphate sodium salt
ATP	Adenosine triphosphate
CD38	NAD^+^ nucleosidase
CD39	Ectonucleoside triphosphate diphosphohydrolase-1
CD73	Ecto-5′-nucleotidase
CFSE	5-carboxyfluorescin succinimidyl ester
eADA	Extracellular adenosine deaminase
ECs	Endothelial cells
EHNA	Erythro-9-(2-hydroxy-3-nonyl)adenine
ENPP1, CD203a	Ecto-nucleotide pyrophosphatase/phosphodiesterase 1
E-selectin	Endothelial selectin
HIF-1α	Hypoxia-inducible factor 1
HMEC-1	Human microvascular endothelial cell line
ICAM-1	Intercellular adhesion molecule 1
INO	Inosine
Jurkat	Human T lymphoblast line, clone E6-1
NAD^+^	Nicotinamide adenine dinucleotide
NADH	Nicotinamide adenine dinucleotide hydrogen
NAM	Nicotinamide
NBTI	S-(4-Nitrobenzyl)-6-thioinosine
NMN	Nicotinamide mononucleotide
NMNAT	Nicotinamide mononucleotide adenylyltransferase
NR	Nicotinamide riboside
NRK1/2	Nicotinamide riboside kinases 1/2
PNP	Purine nucleoside phosphorylase
SIRT1	Sirtuin 1
TAN	Total adenine nucleotides pool
THP-1	Human monocyte cell line
TNF-α	Tumor necrosis factor alpha
VCAM-1	Vascular cell adhesion molecule 1
