# Supplementary material for: NAD+ Enhancer Nicotinamide Riboside Alters Extracellular Purine Metabolism in Human Endothelial Cells
Source: Int J Mol Sci. 2026 Apr 3;27(7):3267. doi: 10.3390/ijms27073267 (PMC13073631; doi:10.3390/ijms27073267)
Supplement: Supplementary file 1 [file ijms-27-03267-s001.zip › ijms-4125241-supplementary.pdf]

Figure S1.

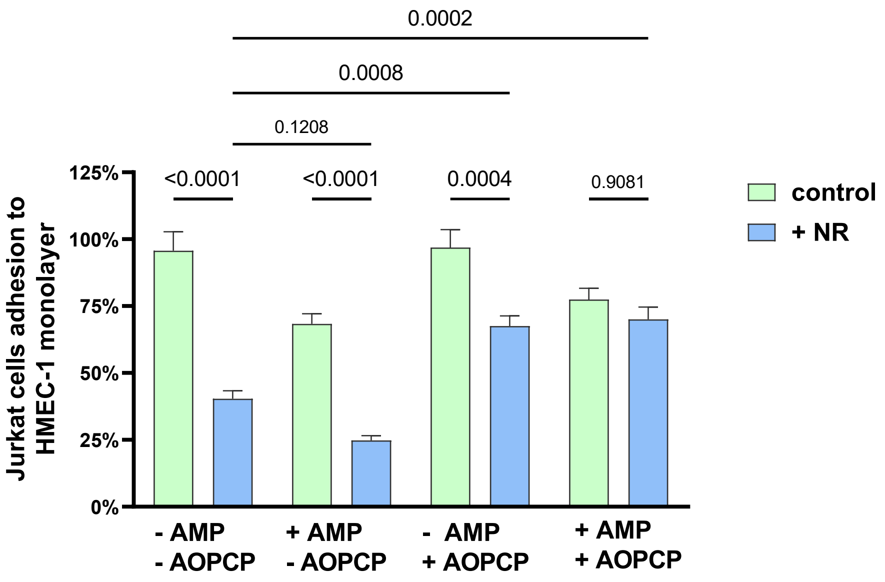

Figure S1. Quantitative analysis of Jurkat cells adhesion to HMEC-1 after co-incubation, expressed as a percentage of the mean number for controls. Results presented as mean ± SEM, *n* = 8-9, one-way ANOVA, Tukey's post hoc test, exact *p*-values shown on the graph.

Figure S2.

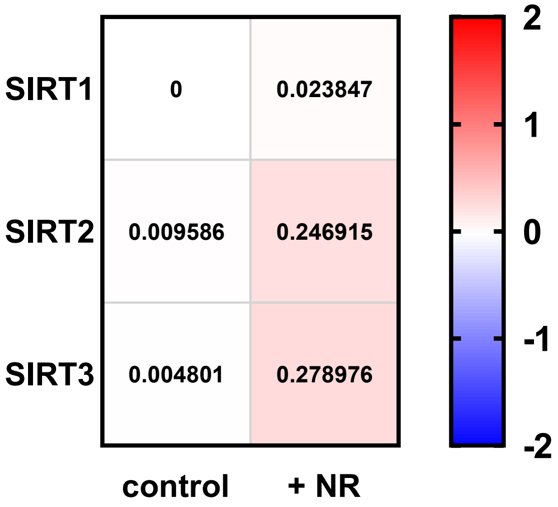

Figure S2. Heatmap illustrating the expression levels of SIRT1, SIRT2 and SIRT3 in control and NR-treated H5V cells. No significant differences in gene expression were observed between the groups. Data are presented as log2-transformed fold changes (2^-ΔΔCt) normalized to control, *n* = 3.

Table S1. Statistical results for all comparisons (*p*, adjusted *p*, t, and df values). *P*-values are provided for comparisons not explicitly indicated in the figures.

Figure			Comparison	control vs + NR, p value	NR vs NR + ENT1i, p value	NR vs NR + PNPi, p value	F (DFn, DFd)	Adjusted P value	t or t,df
Fig 1		a	NAD, H5V	0,003793	0,011005	0,822527	F (3, 16) = 8,147	P=0,001613	
		a	NAD, HMEC-1	0,008556	0,016968	0,976175	F (3, 22) = 6,643	P=0,002312	
		b	NAD/NAD, H5V	0,136363	0,124498	0,987811	F (3, 12) = 3,002	P=0,072663	
		b	NAD/NADH, HMEC-1	0,050497	0,103389	0,17098	F (3, 11) = 2,906	P=0,082569	
		c	NR, H5V	na	0,943602	0,000253	F (3, 9) = 28,75	P=0,000061	
		c	NR, HMEC-1	na	0,900031	0,000016	F (2, 8) = 76,31	P=0,000006	
		d	TAN, H5V	0,942503	0,918697	0,652276	F (3, 22) = 6,643	P=0,002312	
		d	TAN, HMEC-1	0,942265	0,328611	0,857072	F (3, 15) = 1,797	P=0,190869	
		e	ATP/ADP, H5V	0,768789	0,775127	0,768789	F (3, 12) = 1,030	P=0,413948	
		e	ATP/ADP, HMEC-1	0,309131	0,462445	0,031051	F (3, 13) = 3,230	P=0,057618	
		f	AEC, H5V	0,86927	0,930269	0,930269	F (3, 12) = 0,4121	P=0,747362	
		f	AEC, HMEC-1	0,73241	0,73241	0,020237	F (3, 12) = 4,387	P=0,026499	
Fig 2			non-mitochondrial oxygen consumption	0,158499					1,731
			basal respiration	0,231793					1,495
			ATP-linked respiration	0,204712					1,615
			proton leak	0,231129					1,411
			maximal respiration	0,295535					1,202
			spare capacity	0,36285					1,026
			glycolysis	0,6775					1,039
			glycolytic capacity	0,3669					1,703
			glycolytic reserve	0,7662					0,6635
			non-glycolytic acidification	0,7662					0,3809
Fig 3	A	a	1 h	0,155					2,732
			2 h	0,664					1,359
			3 h	0,571					1,521
		b	NAM	0,0009					t=6,083, df=6
		b	ADPR	0,7421					t=0,3447, df=6
		b	AMP	<0,0001					t=6,624, df=6
	B	b	CD38	0,447					t=0,7957, df=9
		b	ENPP1	0,0129					t=2,919, df=12
Fig 4	A	a							t=9,656, df=10

		a							t=4,627, df=9
		b	H5V						t=2,313, df=8
		b	HMEC-1						t=2,665, df=15
		c							t=2,207, df=19
	B	a	H5V						t=9,428, df=33
		a	HMEC-1						t=2,305, df=15
		b							t=2,673, df=10
	C	a	H5V	0,1					t=1,707, df=25
		a	HMEC-1						t=3,162, df=15
		b	ADA1						t=5,323, df=9
		b	ADA2	0,943					t=0,07425, df=6
	D	a							t=8,739, df=13
		b		0,07					t=2,452, df=4
		c		0,6992					t=0,4153, df=4
Fig 5	A	a							t=8,739, df=13
	B	a							t=3,561, df=22
	C	a							
			NR vs control	0,009452				0,009452	F (5, 29) = 7,171
			NR vs NR + CD73i	0,014593				0,014593	
			NR vs NR +. + ARi	0,22979				0,22979	
			NR vs NR + + A_{2A} i	0,947512				0,947512	
			NR vs NR +. + A_{2B} i	<0,0001				<0,0001	

Supplementary Methods

1. Assessment of SIRT1, SIRT2 and SIRT3 genes expression in murine endothelial cells

Total RNA was isolated from H5V cells previously treated with 500 µM NR for 24h, using the RNeasy Plus Universal Mini Kit (QIAGEN Sciences, Germantown, MD, USA) according to the manufacturer's instructions. RNA concentration and purity were assessed using a NanoDrop One spectrophotometer (Thermo Scientific, Waltham, MA, USA). Subsequently, 1.2 µg of total RNA was reverse-transcribed using the Maxima H Minus cDNA Synthesis Master Mix with dsDNase (Thermo Scientific, Waltham, MA, USA).

RNA quality was evaluated using the PrimePCR RNA Quality SYBR Green Assay (Unique Assay ID: qMmuCtlD0001002; Bio-Rad, Hercules, CA, USA). Quantitative real-time PCR reactions were performed using the CFX96 Touch Real-Time PCR Detection System (Bio-Rad, Hercules, CA, USA) with SYBR Green JumpStart Taq ReadyMix (Merck KGaA, Darmstadt, Germany) and gene-specific primers as shown below. Relative mRNA expression levels were calculated using the $2^{-\Delta\Delta Ct}$ method. Values are presented as log2-transformed fold changes ($2^{-\Delta\Delta Ct}$) normalized to control (set as 0). Statistical analysis was performed on ΔΔCt values to assess deviation from 0.

Gene	Starter	Sequence (5'->3')	
SIRT1	forward	TTCAGTGTCATGGTTCCTTTGC	
	reverse	TAGGGCACCGAGGAACTACC	
SIRT2	forward	AGCCAACCATCTGCCACTAC	
	reverse	ATGTGTAGAAGGTGCCGTGG	
SIRT3	forward	GCGTTGTGAAACCCGACATT	
	reverse	GCAAAAGGCTCCACCTCCAG	
	reverse	AAACAGCTCGAAGGAGACGC	
ACTb (Actin Beta)	forward	GGCTCCTAGCACCATGAAGA	internal control
	reverse	GGTGTAAAACGCAGCTCAGTA	
